# The "Medicine in Australia: Balancing Employment and Life (MABEL)" longitudinal survey - Protocol and baseline data for a prospective cohort study of Australian doctors' workforce participation

**DOI:** 10.1186/1472-6963-10-50

**Published:** 2010-02-25

**Authors:** Catherine M Joyce, Anthony Scott, Sung-Hee Jeon, John Humphreys, Guyonne Kalb, Julia Witt, Anne Leahy

**Affiliations:** 1 Department of Epidemiology and Preventive Medicine, Alfred Hospital, Monash University, Melbourne Victoria 3004, Australia; 2 Melbourne Institute of Applied Economic and Social Research, Alan Gilbert Building, The University of Melbourne, Parkville Victoria 3010, Australia; 3 School of Rural Health, Monash University, PO Box 666, Bendigo, Victoria 3552, Australia; 4 Department of Economics, 501 Fletcher Argue Building, University of Manitoba, Winnipeg, MB R3T 5V5, Canada

## Abstract

**Background:**

While there is considerable research on medical workforce supply trends, there is little research examining the determinants of labour supply decisions for the medical workforce. The "Medicine in Australia: Balancing Employment and Life (MABEL)" study investigates workforce participation patterns and their determinants using a longitudinal survey of Australian doctors. It aims to generate evidence to support developing effective policy responses to workforce issues such as shortages and maldistribution. This paper describes the study protocol and baseline cohort, including an analysis of response rates and response bias.

**Methods/Design:**

MABEL is a prospective cohort study. All Australian doctors undertaking clinical work in 2008 (n = 54,750) were invited to participate, and annual waves of data collections will be undertaken until at least 2011. Data are collected by paper or optional online version of a questionnaire, with content tailored to four sub-groups of clinicians: general practitioners, specialists, specialists in training, and hospital non-specialists. In the baseline wave, data were collected on: job satisfaction, attitudes to work and intentions to quit or change hours worked; a discrete choice experiment examining preferences and trade-offs for different types of jobs; work setting; workload; finances; geographic location; demographics; and family circumstances.

**Discussion:**

The baseline cohort includes 10,498 Australian doctors, representing an overall response rate of 19.36%. This includes 3,906 general practitioners, 4,596 specialists, 1,072 specialists in training, and 924 hospital non-specialists. Respondents were more likely to be younger, female, and to come from non-metropolitan areas, the latter partly reflecting the effect of a financial incentive on response for doctors in remote and rural areas. Specialists and specialists in training were more likely to respond, whilst hospital non-specialists were less likely to respond. The distribution of hours worked was similar between respondents and data from national medical labour force statistics. The MABEL survey provides a large, representative cohort of Australian doctors. It enables investigation of the determinants of doctors' decisions about how much, where and in what circumstances they practice, and of changes in these over time. MABEL is intended to provide an important resource for policy makers and other stakeholders in the Australian medical workforce.

## Background

Many countries are experiencing health workforce shortages [1,2]. Medical workforce issues have become increasingly prominent on the national policy agenda in Australia, and have led to significant increases in medical training places[1,3]. Shortages are most pronounced in non-metropolitan areas. To address this, a variety of rural health workforce policies have been implemented in the last decade[4,5]. Career choice by medical graduates is also of increasing interest to policy makers[6]. In the short term, specialties perceived (for various reasons) as being less attractive are experiencing difficulties in recruiting sufficient entrants to their training programs. In the medium term, vastly increased numbers of medical graduates are likely to create different problems for vocational training programs[7].

Research has a key role to play in the development of effective policy responses to challenges such as these. There is increasing recognition of the need for and value of close connections between health services research and health policy, through making policy more evidence-based, and making research more policy-relevant[8,9]. This paper describes a prospective longitudinal study of Australian doctors' workforce participation decisions and their determinants, as the basis for generating evidence of direct relevance in developing more effective medical workforce policies.

Decisions made by doctors about where, when, and how much to work have profound effects on health systems, including equity, capacity, and quality of care. Doctors' labour supply decisions are influenced by a complex mix of factors, including their own preferences about work, leisure, family and lifestyle; economic and non-economic incentives embedded in health system financing and organisation; the culture of medical practice; and longer term trends in demand for health care, demographic change and the composition of the medical workforce.

Existing research has described some of the key trends in Australian medical labour supply, many of which are similar to those in other developed countries. There are notable changes in the gender composition of the workforce, with 34% of Australian doctors in 2006 being female, compared to 18% in 1981[10,11]. Females have comprised approximately 50% of Australian medical graduate cohorts since the late 1990s, and thus the proportion of females in the workforce is expected to continue to rise. This changing gender balance impacts significantly on workforce supply levels, because on average female doctors work fewer hours than male doctors and take more time out from the workforce[10,12]. Workforce participation also varies with age, and the age profile of the Australian medical workforce is changing as well. More than half of the workforce in 2001 were of the 'baby boomer' generation, and therefore are now approaching retirement age [13]. This profile is influenced by historical trends in medical workforce supply policies, with a 'demographic hump' created by the last boom phase in supply growth in the 1970s progressively moving into the older age categories. Graduate numbers in Australia have been cyclic over the decades, with static output levels during the 1980s and 1990s, and the current growth phase commencing around 2000 [14]. This will see graduate numbers increase by 81% between 2005 and 2012[14].

There are a number of important changes associated with these trends, such as decreases in average working hours and differences between cohorts in preferences about workforce participation. Average working hours for female doctors have decreased by 4.6 hours per week (11%) since 1994, while those for male doctors have decreased 9.3 hours per week (17%)[10,11]. This partly reflects cohort effects, with younger doctors of both genders less inclined than their older colleagues to work the long hours traditionally associated with medical practice[13,15]. Another key cohort difference is increased mobility, with younger generations having an increased propensity to move not only between jobs, but also between locations (both within and between nations), and across traditional occupational boundaries[15,16].

While there is a considerable body of research and data describing trends in Australian medical labour supply, there is little research, nationally or internationally, examining the determinants of labour supply decisions for the medical workforce. Although many of these trends are influenced by differences in the preferences of younger cohorts of doctors, they are also influenced by the institutional structures of the health care system, and can therefore potentially be influenced by government policies. This is especially the case for workforce participation, hours worked, workforce distribution, and specialty choice. Although global health workforce shortages suggest that more doctors need to be trained, this is expensive and has very long time lags. There are many other potential policy responses that could increase the productivity of the existing workforce, change the distribution of doctors, or shift tasks to other health workers. Longitudinal data and appropriate statistical analysis are essential for exploring the links between labour supply and its determinants, and large longitudinal data sets covering the entire medical workforce (not just recent graduates) that can be used for this purpose are scarce. The dynamic nature of medical labour markets (with factors such as demand for services, price of services, and government policies playing a role) makes the use of longitudinal data especially important.

We have commenced a longitudinal survey of medical labour market dynamics in Australia, focusing on several key outcome measures including the number of hours doctors work, and decisions to change job, move location, enter a particular medical specialty, or leave the medical workforce. The Medicine in Australia: Balancing Employment and Life (MABEL) study is designed to describe and understand key determinants of these outcomes, including working conditions, job satisfaction, family circumstances, and incentives.

As noted before, the MABEL study was designed to provide research evidence of direct relevance to identified policy issues. The study is advised by a national Policy Reference Group which is a key mechanism to support research-policy linkage. This group comprises representatives from Commonwealth and State government health departments other key organisations such as Rural Workforce Agencies and the Australian Institute of Health and Welfare. The Group helps to ensure that the study is informed by current policy issues and priorities, and to assist in the translation of findings into the policy context.

Implementing a national longitudinal medical workforce study such as MABEL is not without significant challenges. The aim of this paper is to describe the methods and the baseline cohort of the MABEL study. We provide a detailed examination of representativeness, through analysis of response rate and response bias.

## Methods/Design

MABEL is a prospective cohort study of workforce participation and its determinants among Australian doctors. Further information, including copies of the survey instruments, is available on the study's website, http://mabel.org.au. The first wave of data collection, establishing the baseline cohort for the study, was undertaken in 2008, and annual waves of data collection are planned until at least 2011. For the second and subsequent waves, the initial group of responding doctors will be surveyed in addition to a 'top-up' sample that will include all new doctors in the sample frame since the first wave. This will help replace any attrition and maintain the cross-sectional representativeness of each wave.

### Study cohort

Our population of interest is doctors providing clinical medical services in Australia. The most recent estimate of the total medical workforce in Australia (for 2006) was 62,425 doctors, with 58,167 (93%) of these working as clinicians[10]. Within the clinical medical workforce, four broad groups may be distinguished: general practitioners (primary care practitioners); medical specialists; specialists-in-training (vocational trainees or specialist registrars); and hospital non-specialists (including doctors in their early postgraduate years and other hospital doctors not qualified as specialists).

MABEL's sampling frame was the Australasian Medical Publishing Company's (AMPCo) Medical Directory. This national database is used extensively for mailing purposes (e.g. the Medical Journal of Australia). The Directory is updated regularly using a number of sources. AMPCo receives 58,000 updates to doctors' details per year, through biannual telephone surveys and checks medical registration board lists, Australian Medical Association membership lists and Medical Journal of Australia subscription lists to maintain accuracy. The directory contains a number of key characteristics that can be used for checking the representativeness of the sample and to adjust for any response bias in sample weighting. These characteristics include age, gender, location, and job description (used to group doctors into the four types).

At the time of the study's first wave in June 2008 the AMPCo database recorded 58,620 doctors practising in Australia, excluding those known to be not working due to retirement, maternity leave, overseas location or other reasons. Of the 58,620 doctors, 1,552 (2.6%) could not be assigned to one of the four doctor types, either because they did not supply this information to AMPCo (1,261) or because they were not undertaking clinical practice (291). 1,263 doctors (2.2%) did not allow their contact details to be released and 1,059 (1.8%) were non-contactable because they were in the process of having their contact details verified. This left 54,746 doctors in the sampling frame.

Based on results from our piloting, we decided to invite the entire population of doctors rather than select a random sample from the AMPCo list as our sampling frame for MABEL. Our third pilot survey (which included a randomised trial of paper versus online response modes, sampling strategies, and cost-effectiveness analysis), indicated that the census approach was the most cost-effective method to produce a large and representative cohort [17].

Response rate calculations were based on the combined totals from respondents in the third pilot study (February 2008) and those in the main wave (May 2008), as the survey content was very similar. The numerator included respondents to the third pilot and main wave. The denominator included 54,746 from the main wave sample frame from AMPCo in May 2008; plus 35 doctors who were in the sample frame for the third pilot in February 2008 but not in the main wave sample frame in May 2008 (i.e. they were no longer listed in the AMPCo database); less 31 doctors who responded to the first two pilots in October and November 2007. We felt it was not appropriate to send these respondents an invitation to participate in the main wave, due to the short time interval since they completed the pilot study. Thus, the final denominator for the baseline cohort used to calculate the response rate was 54,750.

### Questionnaire design

Study questionnaires went through four stages of piloting. First, the content and face validity of the questionnaire was examined through face-to-face interviews with two to three doctors for each of the four doctor types. Three pilot surveys were administered to random samples of doctors from the AMPCo list (n = 200, 200 and 2,702), helping to ensure that the final versions were as relevant, concise, and clear as possible. The Policy Reference Group provided input to ensure relevance to current policy issues.

The baseline Wave 1 questionnaire included eight sections: job satisfaction, attitudes to work and intentions to quit or change hours worked; a discrete choice experiment (DCE) examining preferences and trade-offs for different types of jobs; characteristics of work setting (public/private, hospital, private practice); workload (hours worked, on-call arrangements, number of patients seen, fees charged; finances (income, income sources, superannuation); geographic location; demographics (including specialty, qualifications, residency); and family circumstances (partner and children).

Job satisfaction was measured using a widely used measure, the ten item version of the Warr-Cook-Wall Job Content Questionnaire [18-20]. The DCE presents a number of paired scenarios describing different job packages and participants are asked, of each pair, which job they prefer. The job packages differ according to a number of predefined job characteristics that might include the earnings, sector of work, hours worked, opportunities for education and training, and characteristics of the work environment. DCEs have been used successfully in examining doctors' preferences for jobs in other studies [21-24].

Different versions of the survey questionnaire were created to tailor the content to the four groups of doctors. A different DCE was administered to each type of doctor. For general practitioners (GPs), the focus was on working in a non-metropolitan area. For non-specialist hospital doctors (the majority of whom are junior doctors in their early postgraduate years), the focus was on speciality choice. For doctors enrolled in a specialty training program and for specialists, the focus was on the balance between public and private sector work. The longest version of the questionnaire (Specialists) contained 87 questions in a 13-page booklet. There is strong evidence that shorter questionnaires yield higher response rates, but in our case it was important to be able to adequately test hypotheses about the potentially wide range of factors that influence labour supply decisions, thus reducing confounding.

### Survey administration

Invitations to participate in MABEL were distributed by mail through AMPCo, in early June 2008. The invitation package included:

• A cover letter on university letterhead using personalised participant information and coloured ink

• A copy of the survey questionnaire, printed in colour

• An explanatory statement providing information about the study, in colour

• A reply-paid envelope[25,26].

• A form to request another version of the survey, for example where the doctor was no longer in a specialist training program but was now a specialist.

Doctors were given the choice of completing a paper copy of the questionnaire or an online version through the secure study website, and were provided with login details in the invitation letter. The content of the online version was identical to the paper version. Participants were able to move forward and backward through the online survey sections and to complete the survey in multiple sessions. Participants completing the online version are presented with a consent form on the log on page for the survey. By logging on, the participant consents to taking part. Participants completing the paper version provide consent by the return of their questionnaire by mail.

In order to be able to draw meaningful inferences about recruitment and retention in rural and remote areas, we needed to ensure a high response rate in these regions, where absolute numbers of doctors are small. Pre-paid monetary incentives, not conditional on response, have been shown to double response rates[25]. Cost considerations precluded use of financial incentives for all participants, but we provided a AU$100 honorarium payment to doctors (mostly GPs) in small rural and remote communities to maximise response rates for this group, in recognition of both their importance from a policy perspective and the significant time pressures doctors in these regions are known to be under[27]. This group was defined by categories 5-7 of the Rural Remote Metropolitan Areas Classification [28].

In the eight months prior to the mail out, the study was widely publicised through direct contact with over 100 medical organisations and medical training colleges and providers. Articles were included in the newsletters and on the websites of many of these organisations. Prior to mail out, MABEL was formally endorsed by over 31 major medical professional organisations such as royal colleges, rural medical groups and medical educational agencies, and this information was included on the cover page of the questionnaire and the study website.

Approximately 4-6 weeks after the initial mailout, a reminder letter was posted to those yet to respond. A copy of the questionnaire was not included with the reminder. The reminder letter was personalised and included an invitation to request another copy of the survey questionnaire, or to log on to the website to complete the survey.

### Ethics approval

The study was approved by the University of Melbourne Faculty of Economics and Commerce Human Ethics Advisory Group (Ref. 0709559) and the Monash University Standing Committee on Ethics in Research Involving Humans (Ref. CF07/1102 - 2007000291).

### Data management and analysis

Data collected through paper questionnaires were entered into an electronic database by a commercial data entry company using double-entry for all variables. A subsequent check of a random sample of 5% of the items entered from the paper questionnaires (352 questionnaires and 64,590 items) found 99.72% accuracy. Additional analysis and cleaning took place to further improve the accuracy of data entry.

Data collected using the online versions of the questionnaires automatically generated a record in an electronic database. These data were downloaded, and data from paper and electronic versions were merged. Standard data checks and cleaning procedures (e.g. range and consistency checks) were used to minimise errors and missing values and to maximise data quality. As noted earlier, all respondents from the third pilot were included in Wave 1.

Data from subsequent waves of the survey will enable more sophisticated analyses which exploit the longitudinal nature of the data on each individual to arrive at more precise and unbiased estimates. Microsimulation models will simulate the effect of changes in determinants such as earnings, taxes, subsidies, job satisfaction, and modifiable job characteristics on key measures of labour supply for the whole population of doctors, providing a valuable resource for national and state workforce planning.

## Discussion

### Response rate

The overall response rate was 19.36% (Table 1). Of the 54,750 doctors who were invited to participate, 0.64% (348) refused to participate, and 0.96% (527) were no longer eligible to participate. With 1,244 mailed questionnaires returned to sender, the overall contact rate was 97.71%. The highest response rate was from specialists (22.34%), followed by specialists-in-training (20.56%), GPs (17.65%) and hospital non-specialists (16.52%). This latter group also had the lowest contact rate, reflecting their higher mobility. A number of doctors fell into a different category to that assigned to them by AMPCo, and so completed a different questionnaire to the one they were initially sent. This may have been due to a change in status (e.g. a specialist-in-training becoming a specialist) or inaccuracies in the AMPCo database. The final numbers of observations available for analysis for each group are therefore different to Table 1: 3,906 GPs, 4,596 specialists, 1,072 specialists in training and 924 hospital non-specialists.

**Table 1:** Response rates

		Doctor type^1^			
		
	All doctors	GP	Specialist	Hospital non-specialist	Specialist in training
a) Total	54,750	22,137	19,579	8,820	4,214
b) Useable responses (with at least one question answered)	10,498	3,873	4,310	1,451	864
c) Refusal (i.e. H/C returned blank, declined)	349	145	124	54	26
d) No contact (return to sender)	1,244	161	307	547	229
e) No responses	42,132	17,762	14,555	6,732	3,083
f) Not eligible (i.e. retired, no longer in clinical practice)	527	196	283	36	12

Response rate (b/(a-f))	19.36%	17.65%	22.34%	16.52%	20.56%
Contact rate ((b+c+e))/(a-f))	97.71%	99.27%	98.41%	93.77%	94.55%
Online responses	30.41%	25.38%	27.60%	47.62%	38.08%

Note 1: Doctor type as defined in the AMPCo database.

Just over 30% of responding doctors chose to fill out the questionnaire online, with the higher proportion of online responders in the younger age groups (specialists-in-training and hospital non-specialists). For the 566 doctors practising in small remote and rural communities who received a cheque with their invite letter, the response rate was 58.44%.

### Response bias

A key issue in survey research is whether respondents differ from non-respondents in some way that is likely to impact systematically on the estimated outcome values. Variables which are of particular relevance to our key outcome variables include age, gender, doctor type, geographic location and hours worked.

Age is a key factor that captures a number of issues including: life-cycle labour supply decisions, such as the decision to raise a family; the propensity of different age cohorts to fill out questionnaires; the 'middle' age ranges being more likely to respond due to lower satisfaction with work and with life in general; and doctors closer to retirement perhaps being less likely to complete the questionnaire because they may regard the survey as less relevant to their situation [29], especially if they are only working a small number of hours in clinical practice.

**Figure 1:**
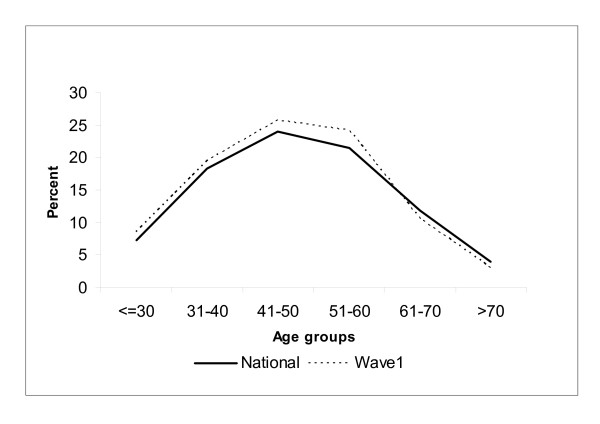
**Age distribution of respondents and population**.

Compared to the total AMPCo population, MABEL has slight over-representation of doctors in all age groups up until age 60 (Figure 1). Respondents aged over 60 years old were slightly under-represented. Overall, the largest difference is observed in the age group 51-60 (21.5% nationally versus 24.2% amongst MABEL respondents).

Table 2 shows differences between the MABEL cohort and the total AMPCo population by doctor type, geographic location and gender. Female doctors are over-represented by six percentage points. Specialists are over-represented by five percentage points, whilst GPs are under-represented by four percentage points. The proportion of hospital non-specialists is lower than in the population, whilst the proportion of specialists-in-training is higher.

**Table 2:** Comparisons of respondent characteristics with population^1^

	National N = 54,750		MABEL respondents N = 10,498	
	**number**	**%**	**number**	**%**
**Doctor type^2^**				
Hospital non-specialists	8,820	16.11	1,451	13.82*
Specialists in training	4,214	7.70	864	8.23*
Specialists	19,579	35.76	4,310	41.06*
GPs	22,137	40.43	3,873	36.89*
**Remoteness (ASGC)^3^**				
Major city	44,623	81.50	8,106	77.21*
Inner regional	7,281	13.30	1,589	15.13*
Outer regional	2,402	4.39	545	5.19*
Remote	349	0.64	207	1.97*
Very remote	95	0.17	51	0.49*
**Gender**				
Male	36,415	66.51	6,392	60.89*
Female	18,308	33.44	4,100	39.06*
Missing	27	0.05	6	0.06

Notes:

1. * p < 0.001. Statistical significance based on a logistic regression model including age, doctor type, gender, and remoteness as independent variables.

2. Doctor type defined by AMPCo, rather than reported in actual survey completed.

3. ASCG = Australian Standard Geographic Classification Remoteness Areas[30].

**Figure 2:**
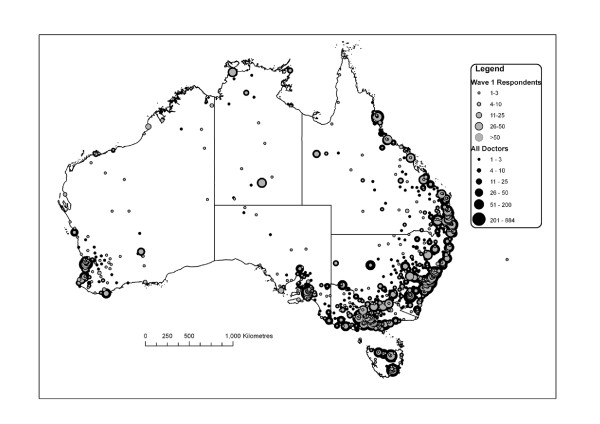
**Geographic distribution of respondents with population of doctors**.

The locality and postcode of doctors' locations of practice were matched to the Australian Standard Geographic Classification Remoteness Areas to compare geographic distribution[30]. Over-representation of doctors in remote and rural areas was anticipated because of the incentive payment. Doctors in major cities were under-represented in our cohort by four percentage points, whilst doctors in all other locations were over-represented, especially remote and very remote areas. Figure 2 shows this on a map of Australia.

Females were over-represented within all doctor types. Over-representation of rural and remote doctors is most marked for GPs but is also apparent to varying degrees for other doctor types. Under-representation of doctors aged 60 and over mainly affects GPs and specialists. Many of the differences in response rates with respect to age, gender, doctor type and location are statistically significant, partly reflecting the large cohort size. The final dataset includes a detailed set of response weights based on a logistic regression model estimated for each doctor type.

**Figure 3:**
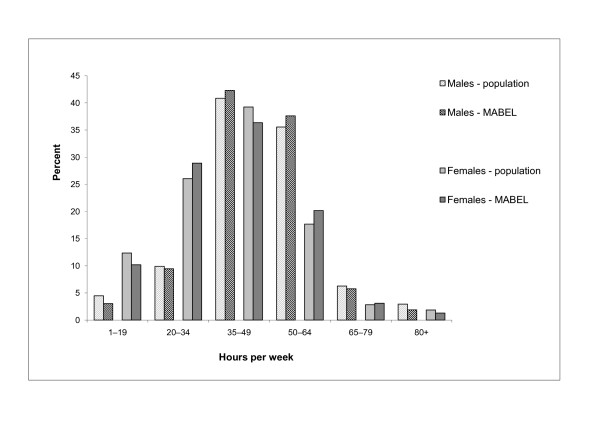
**Comparison of total clinical hours worked per week between respondents and population, by gender**. Source: MABEL and AIHW (2008)[10].

It seems likely that doctors who work longer hours would be less inclined to complete a survey than those who are less pressed for time. In general, the opportunity costs to respondents of filling out the survey are related to the time taken to complete the survey[26,31]. To examine the extent to which MABEL represents doctors who work long hours, we compare the mean and distribution of hours worked with those reported in the Australian Institute of Health and Welfare (AIHW) Medical Labour Force Survey[10]. Table 3 shows differences in the mean of total clinical hours worked weekly. Most differences are less than an hour, with the largest difference for female GPs in MABEL working 1.4 hours more than the population. The distribution of clinical hours worked per week is also similar for MABEL respondents and the population of doctors (Figure 3). For males, there is a slight under-representation at the lower and upper ends of the distribution, and a slight over-representation for males working between 35 and 64 hours per week (around two percentage points). The pattern is more mixed for female doctors, with the largest differences at around three percentage points. The AIHW data on the estimated population of doctors is itself from a registration-based survey with self-reported hours measured using a similar question to that in MABEL.

**Table 3:** Mean total clinical hours worked per week

	Males		*Females*		All doctors	
	MABEL	Population	*MABEL*	*Population*	MABEL	Population
All doctors	47.1	46.6	*38.4*	*37.7*	**43.7**	**43.6**
GPs	45.4	44.2	*33.1*	*31.7*	**39.9**	**39.5**
Specialists	47.1	47	*37.5*	*37.8*	**44.4**	**45**
Hospital non-specialists	49.5	49.4	*45.7*	*45.2*	**47.3**	**47.3**
Specialists in training	50.5	51.7	*47.4*	*46.9*	**48.9**	**49.7**

Source: MABEL and AIHW (2008)[10]

## Conclusion

We have recruited a very large cohort of 10,498 Australian doctors who will be followed prospectively to explore trends in workforce participation and their determinants. This is the largest cohort of Australian doctors in any single research project - cross-sectional or longitudinal. Its size allows us to undertake multivariate statistical analyses, and over time we will draw additional statistical power from the longitudinal aspect of the data.

This study adds to the methodological literature regarding adoption of new technologies in survey research, combining traditional and new methods for contacting potential participants and collecting data. We used posted letters for initial contact, and provided a paper copy of the questionnaire together with the option to use a secure, user-friendly, and flexible online version of the questionnaire.

The study's adoption of explicit mechanisms to connect with the policy context is another notable methodological feature. The Policy Reference Group is a key aspect of this, assisting in translation of findings into the policy context, and ensuring that each wave of the survey is informed by an understanding of current policy issues. Another aspect is the release of de-identified MABEL data for use by other organisations. Release of baseline data is anticipated to occur in early 2010, with annual releases thereafter for subsequent waves. While this is relatively unusual in medical cohort research, it is common for large-scale longitudinal social research studies to make data available to other researchers (e.g., the Household, Income and Labour Dynamics in Australia (HILDA) survey). We believe this will maximise the use of MABEL data and its utility to stakeholders and decision-makers.

Our response rate of 19.4% is at the lower end of the scale for survey research. Published reviews of response rates for medical practitioners in large studies (more than 1,000 respondents) have reported averages of 52%, with a range from 11% to 90%[32]. There is some suggestion that response rates for surveys of medical practitioners are falling[33,34]. Although response rates are used as a 'conventional proxy' for response bias, there is no necessary relationship between response rate and response bias[29,31,35]. Furthermore, despite analysis of response bias being a critical factor in the assessment of sample representativeness, less than half (44%) of published surveys of doctors discuss it and only 18% provide any systematic analysis[32].

Our analyses demonstrate no serious response bias in MABEL with respect to age, gender, geographic location and hours worked. Population data on hours worked are from two years before MABEL. If hours worked were decreasing over that time, then the observed differences may understate the true difference between MABEL respondents and the population. The evidence provides some confidence that we have successfully captured a reasonably representative cohort of doctors across the spectrum of hours worked, including those working long hours.

Our findings accord with literature indicating that response bias is lower in surveys of specific populations, such as medical practitioners, compared to general populations[25,31,32,36]. The existing minor response biases can be adjusted through weighting of analysis. Note that, because we surveyed the entire population, our participants represent not just 19% of the sampling frame but also 19% of the total population. However, we acknowledge that, even if the MABEL cohort is shown to be similar to the population in terms of observed characteristics, there may still be differences in unobserved characteristics that bias responses for some survey questions.

Although many governments and organisations survey doctors, these are usually for census purposes to identify how many doctors there are, where they are practising, and hours of work. Most surveys are not linked over time at the individual level and cannot be used to understand the determinants of labour supply because of their limited range of information. MABEL is a unique longitudinal survey of Australian doctors, focusing on the determinants of labour supply decisions. MABEL will explore workforce dynamics over time, and identify influences on doctors' workforce participation patterns to inform policies for workforce support and management.

## Competing interests

The authors declare that they have no competing interests.

## Authors' contributions

AS, CJ, JH and GK conceived of the study. All authors participated in designing and developing the study instruments and procedures. SHJ performed the data analyses. AS, CJ, and SHJ interpreted the data. CJ and AS drafted the manuscript. All authors read the draft and approved the final version of the manuscript.

## Pre-publication history

The pre-publication history for this paper can be accessed here:

http://www.biomedcentral.com/1472-6963/10/50/prepub
